# Study on the relationship between viral inactivation and alkyl chain length of benzalkonium chloride

**DOI:** 10.1371/journal.pone.0325981

**Published:** 2025-06-30

**Authors:** Ikumi Takada, Atsushi Miyazaki, Chisato Igarashi, Yukiko Yamawaki, Atsuko Hayase, Takuya Mori, Takaya Sakai

**Affiliations:** 1 Biological Material Science Research Laboratory, Kao Corporation, Tochigi, Japan; 2 Biological Material Science Research Laboratory, Kao Corporation, Tokyo, Japan; 3 Household Research Laboratory, Kao corporation, Wakayama, Japan; 4 Biological Material Science Research Laboratory, Kao Corporation, Wakayama, Japan; 5 Research and Development, Kao Corporation, Tokyo, Japan; Universidad Autonoma de Chihuahua, MEXICO

## Abstract

Alkyldimethylbenzylammonium chloride (BAC), a quaternary ammonium salt surfactant, is commonly utilized as a sanitizer and disinfectant. Concerns have emerged regarding the increasing global exposure to BAC, emphasizing the need for strategies to maximize its efficacy while minimizing its usage. Therefore, it is crucial to understand BAC’s effects on bacteria and viruses, and its mechanism of action. Our previous research demonstrated that BAC micelles solubilizes the lipid bilayer of viruses, significantly enhancing virus inactivation efficacy at concentrations exceeding the critical micelle concentration (CMC). We focused on the alkyl chain length of BAC to elucidate how variations in CMC, driven by differences in alkyl chain length, influence virus inactivation activity. We measured CMC and assessed influenza virus inactivation for benzyldodecyldimethylammonium chloride (C12BAC), benzyltetradecyldimethylammonium chloride (C14BAC), benzylhexadecyldimethylammonium chloride (C16BAC), and their mixtures. The results showed that both BACs with a single alkyl chain length and mixed BACs exhibited significantly enhanced inactivation activity at concentrations of the CMC or above. Notably, BAC mixtures with comparable CMC values showed similar virucidal activity, suggesting that CMC can serve as a useful indicator in designing BAC mixtures aimed at virus inactivation, leading to the development of compositions with enhanced virus inactivation at lower doses. However, when the bactericidal activity of each BAC mixture against *E. coli* was tested under the same conditions, a significant bactericidal effect, of at least 3 Log_10_, was observed even at concentrations below CMC. This suggests that the bactericidal activity of BAC is not due to the micellization at CMC, but rather to the contribution of BAC monomers, indicating a distinct difference in the mechanism of action of BAC against viruses and bacteria. Therefore, when formulating BAC-based disinfectants, it is essential to assess the inactivation efficacy against both bacteria and viruses to ensure sufficient virus inactivation.

## Introduction

The COVID-19 caused a global outbreak and has had a profound impact on both society and the global economy. Humanity has faced, similar to COVID-19, epidemics and pandemics in the past, including the Spanish flu, Middle East respiratory syndrome (MERS), and the 2009 H1N1 influenza. As a result, many scientists urge caution against the possibility of future pandemics caused by unknown pathogens. Infection prevention measures are critical, with the proper use of hand sanitizers and environmental disinfectants being among the key strategies [1,2]. While ethanol is commonly used as the active ingredient in disinfectants, frequent use of ethanol-based sanitizers can cause skin irritation and roughness [3]. Additionally, concerns exist about the flammability of high-concentration ethanol in environmental disinfectants. Consequently, in daily life, long-chain alkyl quaternary ammonium salts, a type of surfactant, are often used as active ingredients in non-alcoholic hand sanitizers and environmental disinfectants.

One of the most commonly used long-chain alkyl quaternary ammonium salts is alkyldimethylbenzalkonium chloride (BAC). BAC serves a variety of functions, including as a disinfectant, preservative, and solubilizer, and its usage is predicted to increase [4]. However, concerns have been raised regarding the environmental impact of increased BAC usage, and it is supposed that environmental monitoring of BAC is necessary [5,6]. To support the goal of a sustainable society, it is essential to optimize BAC use by ensuring its effectiveness with minimal quantities.

BAC is recognized for its potent bactericidal activity [7,8], with its mechanism of action involving the adsorption of positively charged BAC molecules to the negatively charged bacterial cell membrane, leading to membrane disruption [9,10]. In contrast, the effect of BAC on enveloped viruses is less consistent, with some studies reporting an inactivating effect while others show no impact [11,12]. It has been proposed that surfactants like BAC may inactivate viruses by disrupting the viral envelope at concentrations of the critical micelle concentration (CMC) or above, although the precise mechanism of this disruption remains unclear [13–16]. The CMC is defined as the concentration at which a surfactant transitions from monomeric state to forming micelles in aqueous solution, indicating the solubility threshold for surfactant molecules. Our previous research has shown that the mechanism by which BAC inactivates viruses is dependent on the CMC. Specifically, below the CMC, virus inactivation occurs through the adsorption of BAC monomers. However, at the CMC or above, in addition to monomer adsorption, the lipid molecules in the viral envelope are solubilized into micelles, leading to membrane collapse and a marked increase in virus inactivation efficacy [17]. Moreover, we demonstrated that the CMC of BAC varies with the salt concentration in the virus reaction solution, altering the concentration range at which BAC exhibits virus inactivation activity [17].

The CMC is influenced by several factors, including salt concentration, pH, and temperature. One key factor affecting the CMC of a surfactant is the alkyl chain length of BAC [18]. Typically, BAC is present as a mixture of linear alkyl chains with 12–16 carbon atoms (C12-C16). However, there have been no detailed studies examining the impact of BAC’s alkyl chain length or the composition of alkyl chain mixtures on the inactivation of enveloped viruses. Thus, in this study, we investigated how variations in BAC alkyl chain length affect its inactivation activity against influenza virus (IFV), a representative enveloped virus. The results provide design guidelines for BAC compositions to achieve the desired virus inactivation for use in hand sanitizers and environmental disinfectants, ultimately contributing to a reduction in BAC usage.

## Results

### CMC and virucidal activity of BAC with single alkyl chain length

**Fig 1:**
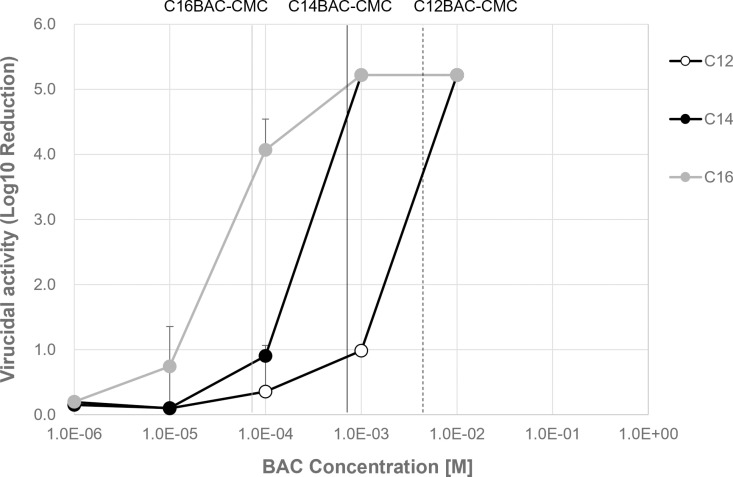
Virucidal activity of BAC with single alkyl chain. IFV(1 x 10^9^ FFU/mL) with SFM and BAC were contacted in 30 min (IFV:BAC = 1:9). Log_10_ Reduction was calculated from the virus titer. White circle: C12BAC; black circle: C14BAC; gray circle: C16BAC (Mean ± SD, n = 3).

Aqueous solutions of 10 ⁻ ⁶, 10 ⁻ ⁵, 10 ⁻ ⁴, 10 ⁻ ³, and 10 ⁻ ² M of dodecyldimethylbenzalkonium chloride (C12BAC), tetradecyldimethylbenzalkonium chloride (C14BAC), and hexadecyldimethylammonium chloride (C16BAC), each containing linear alkyl chains of C12, C14, and C16, respectively, were prepared. These solutions were then exposed to a virus solution of 1 × 10⁹ FFU/mL for 30 min to assess their virucidal activity. The results showed a log10 reduction of upper detection limit value of 5.22 for C12BAC at 10 ⁻ ² M, 5.22 for C14BAC at concentrations of 10 ⁻ ³ M or higher, and 4.07 for C16BAC at 10 ⁻ ⁴ M, with a 5.22 log10 reduction of virus titer observed at 10 ⁻ ⁴ M or higher (Fig 1, S1 Table). Additionally, the CMCs of BACs with different alkyl chain lengths were measured in a solution containin10% serum-free medium (SFM), under the same solvent conditions used for the virucidal evaluation. The CMCs for C12BAC, C14BAC, and C16BAC were determined to be 4.54 × 10 ⁻ ³ M, 7.27 × 10 ⁻ ⁴ M, and 7.43 × 10 ⁻ ⁵ M, respectively (Table 1). These findings demonstrate that the virucidal activity of BACs with varying alkyl chain lengths is significantly enhanced at concentrations of their respective CMCs or above.

**Table 1:** Critical micelle concentration (CMC) of BAC with single alkyl chain.

Alkyl chain length	CMC [M]
C12	4.54E-03
C14	7.27E-04
C16	7.43E-05

CMC was measured in the BAC aqueous solution with 10% SFM (the same condition as virus activity evaluation).

### CMC and virucidal activity of BAC with some kinds of alkyl chain length

**Fig 2:**
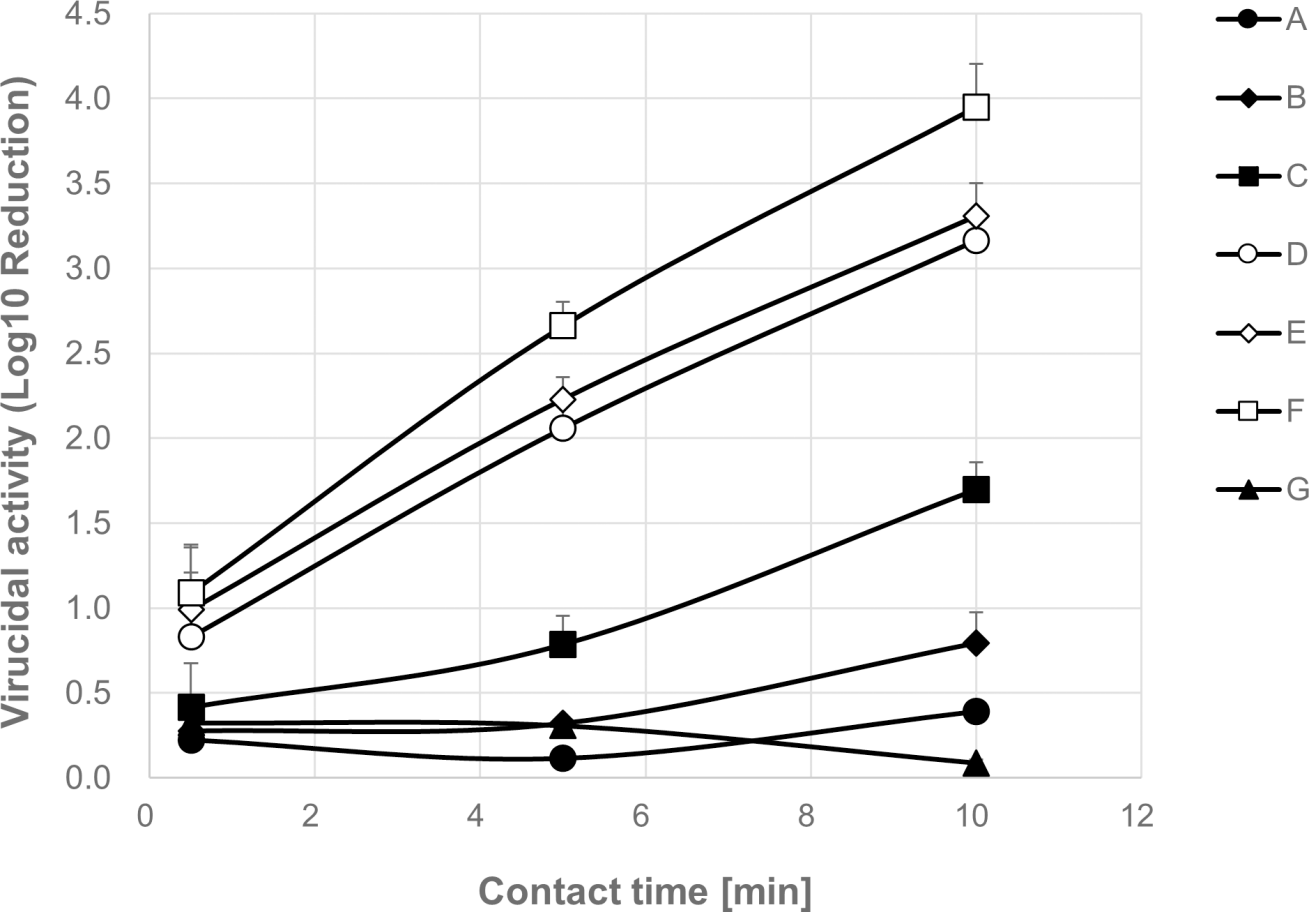
Virucidal activity of BAC with chain lengths of C12, C14 and C16. IFV (1 x 10^9^ FFU/mL) with SFM and BAC (10^−4^ M) were contacted in the optional time (IFV:BAC = 1:9). Log_10_ Reduction was calculated from the virus titer (Mean ± SD, n = 3).

**Fig 3:**
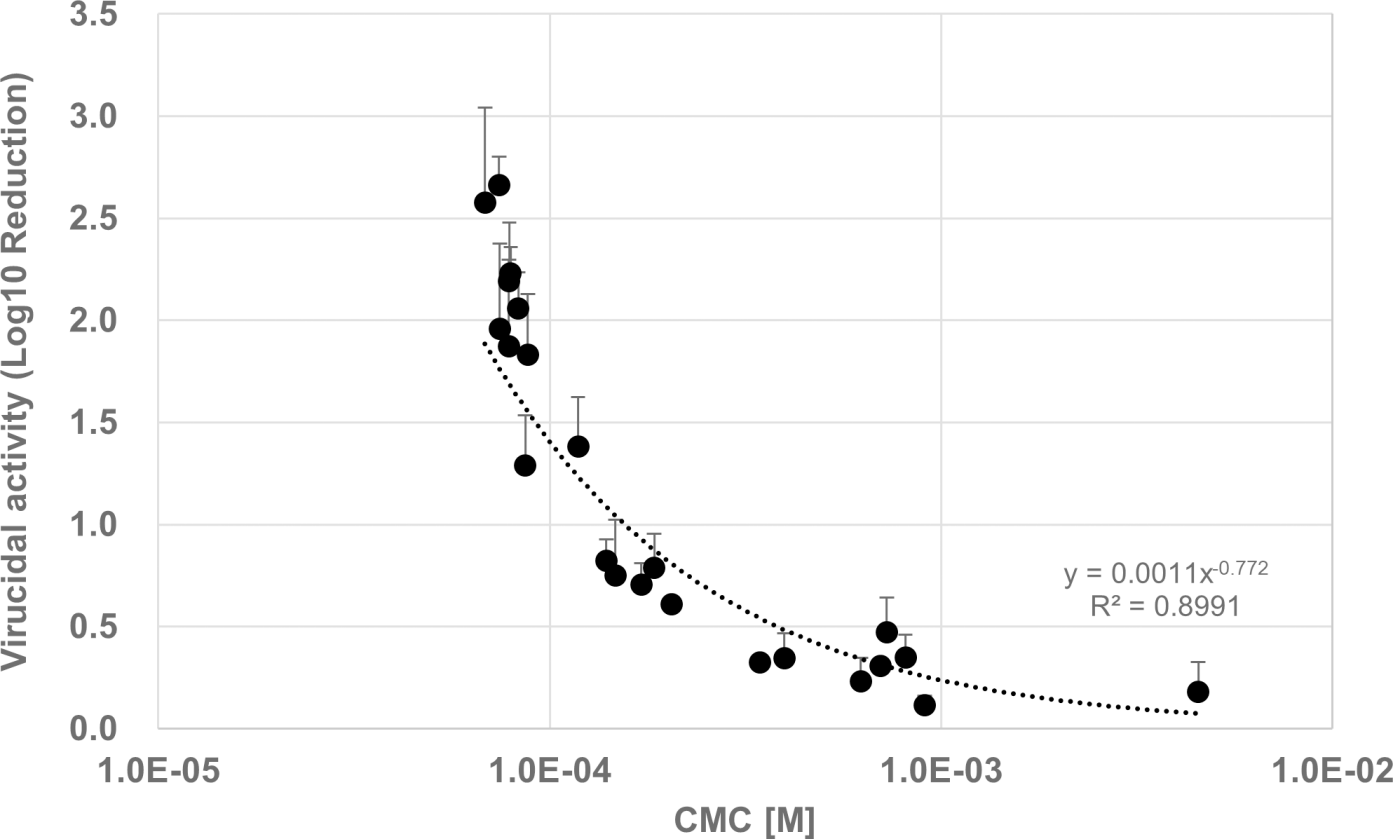
Virucidal Activity and CMC of BACs with different CMC. IFV (1 x 10^9^ FFU/mL) with SFM and BAC (10^−4^ M) were contacted in 5 minutes (IFV:BAC = 1:9). Log_10_ Reduction was calculated from the virus titer (Mean ± SD, n = 3).

C12BAC, C14BAC, and C16BAC were combined in various ratios to prepare six different BAC composition solutions (Table 2 A-F). The CMC of each composition solution was measured, revealing that a higher ratio of C16BAC corresponded to a lower CMC value (Table 2). Based on these CMC values, the evaluation concentration was set at 10 ⁻ ⁴ M, with half of the compositions having concentrations below the CMC and the other half above the CMC. The virucidal activity of each BAC composition solution was then assessed over time. Results showed that, 5 and 10 min after the start of the virucidal reaction, the log10 reduction in virus titer was 0.11/0.39 for composition A, 0.31/0.79 for composition B, 0.79/1.70 for composition C, 2.06/3.16 for composition D, 2.23/3.31 for composition E, and 2.66/3.95 for composition F (Fig 2, S2 Table). The commercially available reagent G demonstrated a log10 reduction of 0.31/0.09 at 5/10 min. In other words, compositions D, E, and F (Fig 2, open plots), with the CMC below the evaluation concentration of 10 ⁻ ⁴ M, achieved a log10 reduction of 2 or more in virus titer within 5 min of the reaction. Conversely, compositions A, B, C, and G (Fig 2, solid plots), with the CMC above the evaluation concentration of 10 ⁻ ⁴ M, did not achieve a 2 log_10_ reduction in virus titer within 5 min and required more than 10 min to reach this level. Additionally, several BAC compositions with varying ratios of C12BAC, C14BAC, and C16BAC were prepared, and both virus inactivation evaluation and CMC measurements were conducted. Compositions (H, I, J, K, L, M) with the CMC below the evaluation concentrations of 10 ⁻ ⁴ M demonstrated high virus inactivation activity (S2 Table). Conversely, compositions (N, O, P, Q, R, S, T, U, V, W) with the CMC above 10 ⁻ ⁴ M exhibited lower activity. A lower CMC correlated with higher virus inactivation activity in the compositions (Fig 3). Interestingly, BACs with similar CMCs exhibited comparable virus inactivation activity, irrespective of the alkyl chain composition ratio. These findings indicate that BACs achieve high virus inactivation activity, when the evaluation concentration is equal to or exceeds the CMC, implying the presence of BAC micelles under the evaluation conditions is important for virucidal activity.

**Table 2:** CMC of BAC with chain lengths of C12, C14 and C16.

Sample	C12:C14:C16 composition (Molar ratio)	CMC [M]
A	1:1:0	9.09E-04
B	1:1:1	3.44E-04
C	1:1:3	1.85E-04
D	1:1:8	8.31E-05
E	1:1:18	7.95E-05
F	0:0:1	7.43E-05
G	61:32:7	7.01E-04

CMC was measured in the BAC aqueous solution with 10% SFM (the same condition as virus activity evaluation).

### Bactericidal activity of BAC with some kinds of alkyl chain length

**Fig 4:**
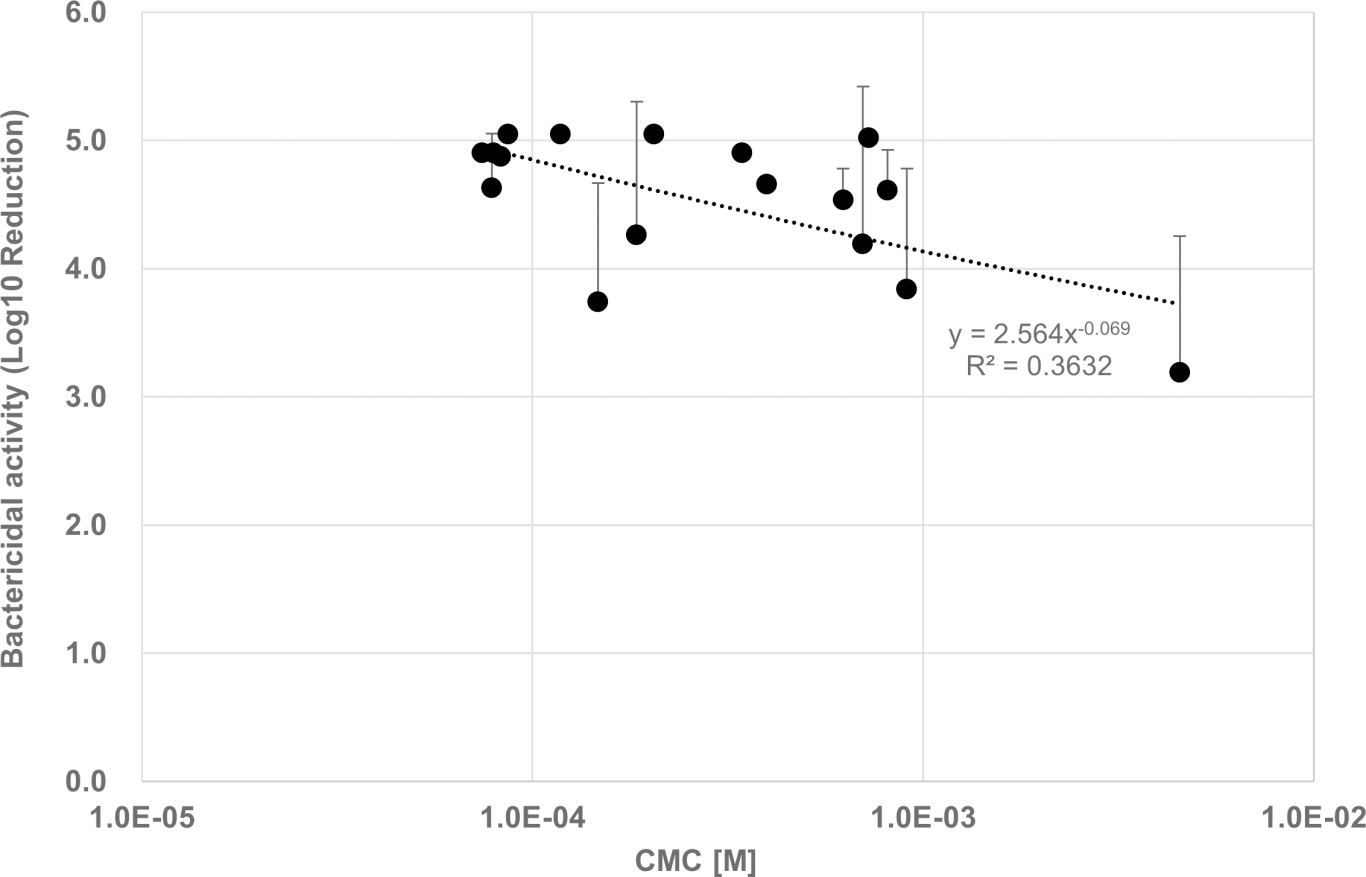
Bactericidal Activity and CMC of BACs with different CMC. *E. coli* (1 x 10^9^ CFU/mL) with D-PBS and BAC(10^−4^ M) were contacted in 5 minutes (*E. coli*:BAC = 1:9, Mean ± SD, n = 3).

The bactericidal activity of a mixture of C12BAC, C14BAC, and C16BAC against *E. coli* was assessed under the same conditions (22°C, 5 min) as the virus inactivation activity evaluation described previously. Given that the CMC of C12BAC dissolved in a solution containing 10% SFM and dissolved in a solution containing 10% PBS are nearly identical [17], D-PBS was used as the suspension medium for *E. coli* in the bactericidal activity evaluation. The composition with the lowest activity, composition W, demonstrated a 3.19 log reduction, while other compositions also exhibited high activity (S2 Table). It was found that the bactericidal activity by BAC is not affected by whether its concentration exceeds the CMC or not (Fig 4).

## Discussion

In this study, we investigated the relationship between the alkyl chain length of BAC and its virucidal activity by measuring the CMC and evaluating influenza virus inactivation for C12BAC, C14BAC, C16BAC, and their mixtures. The CMC depended on the alkyl chain length of the BAC, but the virucidal activity of any BAC varies significantly depending on whether the concentration exceeds the CMC, showing high virucidal efficacy when above the CMC (Figs 2 and 3). Additionally, BACs with similar CMCs demonstrated comparable virucidal activity, regardless of the alkyl chain composition (Fig 3). These results indicate that exceeding the CMC is essential for achieving sufficient virus inactivation.

Surfactants with longer alkyl chains are known to be more hydrophobic and exhibit lower CMC values [19–21]. In this study as well, we observed that, as the chain length of single alkyl chain BACs increased, or as the proportion of C16BAC in mixed compositions increased, the CMC decreased (Tables 1,2 and S2). It has been reported that the virucidal activity of C12BAC significantly improves at concentrations above the CMC [17]. The CMC represents the concentration at which a surfactant’s dissolved state in aqueous solution undergoes a significant change, marking the solubility limit concentration of surfactant monomers. Below the CMC, surfactants exist as monomers, whereas above the CMC, excess surfactant forms aggregates (micelles) consisting of multiple monomers, with both monomers and micelles coexisting. Micelles can solubilize hydrophobic molecules that are insoluble in water. Therefore, the virucidal mechanism of BAC at concentrations of the CMC or above is proposed to involve not only the weakening of the viral envelope membrane due to monomer adsorption but also the solubilization of envelope membrane lipids by micelles [17]. This study demonstrates that the alkyl chain length of the components in BACs is an important factor that affects the CMC, and that virucidal reactions progress very little even at high concentrations if the CMC is not exceeded. It confirms that the CMC has a significant impact on virucidal activity. These findings support previous research [17].

In contrast, the bactericidal activity against *E. coli* was evaluated under the same conditions, but unlike virucidal activity, high bactericidal activity was observed even at concentration below the CMC (Fig 4). Previous studies have indicated that variations in the alkyl chain length of BACs impact their bactericidal and antibacterial activities [22–25]. Specifically, BACs with alkyl chain lengths of C12, C14, and C16 demonstrate consistently higher bactericidal activity against *E. coli* compared to BACs with chain lengths of C10 or shorter or C18 or longer [9,23,26,27], although the relationship with the CMC was not clearly defined. The results of this study support these findings, demonstrating that bactericidal activity does not depend on the CMC. Additionally, it has been reported that the minimum bactericidal concentration (MBC) of various quaternary ammonium salts against *E. coli* is 10–700 times lower than the CMC [28]., suggesting that the MBC for the compositions used in this evaluation is at least 10 times lower than the CMC. These findings suggest that effective bactericidal activity requires an appropriate chain length and not micellization.

Based on the results of this study and previous findings, it can be inferred that the mode of action of BAC differs between viruses and bacteria. The action of BAC on envelope viruses with a vesicle structure can be explained simply in terms of physical science, but bacteria are complex, so the effects on bacteria cannot be easily explained like those on viruses. The major factor contributing to this difference seems to be the system of life activities of bacteria and enveloped viruses. Bacterial outer membranes consist of both a cell membrane and a cell wall. While the cell wall structures of gram-negative and gram-positive bacteria differ, the bactericidal effect of BAC is reportedly similar for both types [22]. The bactericidal mechanism involves BAC adsorption to the bacterial cell membrane, leading to membrane destruction and the release of intracellular components such as proteins and nucleic acids, with autolysis occurring at concentrations lower than those required for bactericidal activity [12,29]. Additionally, BAC-induced membrane damage has been associated with morphological changes like the formation of protrusions and blisters on bacterial surfaces [29,30]. In contrast, the virucidal mechanism for envelope viruses including influenza viruses appears to depend on the BAC concentration. Below the CMC, BAC monomers adsorb to the viral surface and inactivate it, while at the CMC or above, BAC micelles solubilize the viral envelope membrane, leading to virus particle destruction [17]. Therefore, when using BAC as an active ingredient in disinfectants, it is crucial to evaluate its effectiveness against both bacteria and viruses, given the differing inactivation mechanisms for these pathogens.

A limitation of this study is the restricted range of alkyl chain lengths evaluated for BACs. We focused on BACs with the commonly used chain lengths of C12, C14, and C16. Preliminary research indicated that C10 BACs did not exhibit virus inactivation activity (S3 Table), and C18 BACs precipitated rather than dissolving under test conditions, rendering them unsuitable for CMC discussions. Although longer chain lengths generally increase inactivation activity for many bacteria and yeasts, improvements plateau beyond a certain chain length due to solubility issues [31]. It is crucial to explore reaction conditions that adequately assess the relationship between CMC and virus inactivation activity for BACs with a broader range of alkyl chain lengths and their mixtures. Another limitation is that the concentrations used in the evaluation of BAC are limited. It is necessary to determine the minimum bactericidal concentration (MBC) and minimum viral inactivation activity, and to analyze their relationship with CMC in order to explore the mechanisms in more detail.

BAC is typically used in hand sanitizers and environmental disinfectants at concentrations of 10^−3^ M (approximately 0.05 w/w%) or higher. In this study, all BAC compositions evaluated demonstrated high virus inactivation activity at 10^−3^ M (S2 Table). Conversely, at the 1/10 concentration of 10^-4^M, the virucidal activity varied depending on the composition, with only certain BAC compositions exceeding the CMC demonstrating higher virucidal activity than commercially available reagents. Additionally, it was suggested that the required dissolved state of BAC differs between its effects on bacteria and viruses. These results indicate the necessity of considering the differences in inactivation mechanisms for the target pathogens when using BAC as an active ingredient in disinfectants and evaluating its efficacy. Furthermore, in the future, optimizing BAC compositions using CMC as a guiding parameter is anticipated to enable efficient bactericidal and virucidal activity at lower concentrations. This approach is anticipated to be valuable for developing BAC formulations that are both highly effective and environmentally and safety concerns.

## Conclusion

This paper explored the relationship between virus inactivation activity and CMC using BACs with various alkyl chain lengths. Evaluating different mixed chain length BACs demonstrated that a lower CMC correlates with higher virus inactivation activity, underscoring the critical role of CMC in BAC-mediated virus inactivation. The study’s findings enable the proposal of specific alkyl chain compositions for BACs that achieve high virus inactivation activity at lower concentrations, which is anticipated to facilitate the development of BAC formulations with improved environmental and safety profiles. Furthermore, since conditions satisfied with both bactericidal activity and virus inactivation are different, it is essential to verify its efficacy against both bacteria and viruses when considering its use as a disinfectant.

## Materials and methods

### Reagents

Benzyldodecyldimethylammonium chloride (C12BAC) and benzyldimethyltetradecylammonium chloride (C14BAC) were obtained from Tokyo Chemical Industry Co., Ltd., while Cetalkonium chloride (C16BAC) was sourced from TRC Toronto Research Chemicals Inc. Mixed chain length BACs were prepared by combining these BACs in various molar ratios. For a commercially available reagent, a 10% Benzalkonium Chloride Solution (Fujifilm Wako Pure Chemical Corporation) was used as the reagent. After diluting to the evaluation concentration, the solution was stored at −20°C for one week or more, then brought back to room temperature for use in the experiments.

### Cell, virus and bacteria

MDCK cells (CCL-34) were obtained from the American Type Culture Collection (ATCC) and were cultured in Eagle’s Minimum Essential Medium (MEM, Invitrogen) supplemented with 5 v/v% immobilized fetal bovine serum (FBS, Sigma-Aldrich) and 50 µg/mL gentamicin sulfate (Invitrogen) at 37°C under 5% CO_2_.

Influenza virus A/PR/8/34 (H1N1, VR1469) was also sourced from the ATCC. MDCK cells were infected with the virus at a multiplicity of infection (MOI) of 0.001 and cultured in serum-free medium (SFM, Invitrogen) supplemented with 1 µg/mL trypsin (Sigma-Aldrich) and 50 µg/mL gentamicin sulfate at 37°C under 5% CO_2_ for 48 h. The culture supernatant was collected, centrifuged at 800 g for 10 min at 4°C to remove impurities, and then further centrifuged at 13,000 g for 2 h at 4°C to obtain a concentrated virus pellet. This pellet was resuspended in SFM, further centrifuged at 800 g for 10 min at 4°C, and the supernatant was collected to yield a concentrated virus solution.

*Escherichia coli* (*E. coli*, NBRC 3301) was purchased from the NITE Biological Resource Center (NBRC). The bacteria were cultured on SCD (Soybean-Casein Digest) agar medium (AS ONE Co., Ltd.) at 37°C. The cultured *E. coli* was transferred to SCD liquid medium (Nihon Pharmaceutical Co., Ltd.) using a platinum loop and incubated overnight at 37°C with shaking at 200 rpm for use in the experiments.

### Measurement of CMC

In accordance with methodology given in a previous report [17], the CMC was calculated from (A) surface tension measurements and (B) dye solubilization measurements, and their good agreement was confirmed. BAC was dissolved in an aqueous solution containing 10% serum-free medium (SFM) for the CMC measurements.

**(A) Surface tension measurements**

The surface tension of the BAC aqueous solutions was measured at 25°C using Wilhelmy Pt plate technique with a tensiometer K100 MK2 (Krüss GmbH). The test solutions were placed in a Teflon petri dish with a lid and stood in a thermostatic chamber at 25°C for at least one day and night before measurement. The surface tension was taken after measurement fluctuation was less than 0.1 mNm-1 for 1 hr. The concentration at which the concentration-surface tension curve bends was determined as the CMC.

**(B) Solubilization of oil-soluble dye**

Five mL of test solution and the required amount of oil-soluble dye, 1-(o-Tolylazo)-2-naphthol, were mixed in a vial. The vial was then gently shaken for a few days in a water bath at 25°C, followed by filtering to remove the insoluble excess dye with a disposable filter unit DISMIC-13P (Advantec Toyo Kaisha, Ltd). Four mL portion of ethanol was added to 1 mL of the filtrate and the absorbance of the solution was measured at 489 nm using a UV-vis spectrophotometer (V-700, Jasco Corporation) to determine the amount of solubilized dye. The inflection point of the absorbance curve was assumed to be the CMC.

### Evaluation of virucidal activity

In accordance with ASTM-E1052, 45 µL of water (control) or BAC solution was mixed with 5 µL of influenza virus solution (1.0 × 10^9^ FFU/mL) at 22°C for a specified duration. The reaction was halted by adding 950 µL of SCDLP (Soybean-Casein Digest Broth with Lecithin and Polysorbate 80) medium (Nihon Pharmaceutical Co., Ltd.). The virus titer was assessed with minor modifications based on previous reports [32–35]. Specifically, the virus solution obtained from the inactivation evaluation was diluted with SFM and used to infect confluent MDCK cells cultured in a 12-well plate for 30 min. After infection, the plate was washed with SFM, and 2 mL/well of SFM supplemented with 1.2% cellulose (CEOLUS, Asahi KASEI), 2 µg/mL trypsin, and 50 µg/mL gentamicin sulfate was added. The plate was then incubated at 37°C for 18–22 h. Following incubation, the cells was fixed with ice-cold ethanol (Fujifilm Wako Pure Chemical Corporation). Cells were then stained with a mouse anti-N-Protein antibody as the primary antibody and HRP-conjugated goat anti-mouse IgG + IgM (Jackson ImmunoResearch) as the secondary antibody. The virus concentration was expressed as focus-forming units per mL (FFU/mL). The Log_10_ FFU/mL value was calculated, and the difference from the Log_10_ FFU/mL value of the control was expressed as Log_10_ reduction (virus inactivation/virucidal activity value). The virucidal activity was evaluated after confirming the neutralizing activity using SCDLP in advance.

### Evaluation of bactericidal activity

The *E. coli* culture was centrifuged at 10,000 rpm for 1 min, washed with D-PBS(-), and resuspended in D-PBS(-) to prepare a bacterial solution with 3.0 of an OD600 (1.0 × 10^9^ CFU/mL). Subsequently, 45 µL of water (control) or BAC solution was mixed with 5 µL of the *E. coli* solution at 22°C for 5 min. The reaction was terminated by adding 950 µL of SCDLP medium.

Bactericidal activity was assessed with minor modifications based on a previous report [36]. The stopped bacterial solution was mixed with SCD medium, and the activity value was determined from the growth curve monitored using a BioTek LogPhase 600 microbiology plate reader (Agilent Technologies, Inc.). Additionally, a mixture of 45 µL of pure water, 5 µL of bacterial solution, and 950 µL of SCDLP medium (Standard) was inoculated onto SCD agar medium and incubated overnight at 37°C. Viable bacteria counts were performed, and the number of viable bacteria in each sample was calculated from the standard viable bacteria count and the activity value obtained using the BioTek LogPhase 600 microbiology plate reader.

## Supporting information

S1 Table Virus titer and Log_10_ Reduction of BAC with single alkyl chain. Log_10_ Reduction is the difference between virus titer of each BAC and the control (Mean ± SD, n = 3).
(DOCX)

S2 Table Virucidal and Bactericidal activity of BAC mixture with alkyl chain lengths of C12, C14 and C16. Log_10_ reduction value was calculated based on the difference in viral titers and viable cell counts following treatment with the control and BAC mixture. (Mean ± SD, n = 3–6). Composition G is a commercially available reagent. “n.t.” indicates not tested, “-“ denotes that no calculable values are available, and “LR” indicates Log Reduction value.
(DOCX)

S3 Table Virus titer and Log_10_ Reduction of C10 BAC. Log_10_ Reduction is the difference between virus titer of each BAC and the control (Mean, n = 2).
(DOCX)
