# Supplementary material for: Study on the relationship between viral inactivation and alkyl chain length of benzalkonium chloride
Source: PLoS One. 2025 Jun 30;20(6):e0325981. doi: 10.1371/journal.pone.0325981 (PMC12208410; doi:10.1371/journal.pone.0325981)
Supplement: S1 Table — Log10 Reduction is the difference between virus titer of each BAC and the control (Mean ± SD, n = 3). (DOCX) [file pone.0325981.s001.docx]

**S1 Table. Virus titer and Log_10_ Reduction of BAC with single alkyl chain**

| **Sample** | **Concentration [M]** | **Virus Titer [FFU/mL, Log]** | | | **Log Reduction** |
| --- | --- | --- | --- | --- | --- |
| Control |  | 6.52 | ± | 0.12 |  |
| C12 BAC | 10^-2^ | 1.30 | ± | 0.00 | 5.22 |
|  | 10^-3^ | 5.53 | ± | 0.24 | 0.99 |
|  | 10^-4^ | 6.16 | ± | 0.07 | 0.35 |
|  | 10^-5^ | 6.42 | ± | 0.15 | 0.10 |
|  | 10^-6^ | 6.32 | ± | 0.02 | 0.20 |
| C14 BAC | 10^-2^ | 1.30 | ± | 0.00 | 5.22 |
|  | 10^-3^ | 1.30 | ± | 0.00 | 5.22 |
|  | 10^-4^ | 5.62 | ± | 0.17 | 0.90 |
|  | 10^-5^ | 6.41 | ± | 0.00 | 0.10 |
|  | 10^-6^ | 6.37 | ± | 0.06 | 0.15 |
| C16 BAC | 10^-2^ | 1.30 | ± | 0.00 | 5.22 |
|  | 10^-3^ | 1.30 | ± | 0.00 | 5.22 |
|  | 10^-4^ | 2.45 | ± | 0.47 | 4.07 |
|  | 10^-5^ | 5.78 | ± | 0.61 | 0.74 |
|  | 10^-6^ | 6.32 | ± | 0.05 | 0.20 |

Log_10_ Reduction is the difference between virus titer of each BAC and the control (Mean ±SD, n = 3)
