# Supplementary material for: Study on the relationship between viral inactivation and alkyl chain length of benzalkonium chloride
Source: PLoS One. 2025 Jun 30;20(6):e0325981. doi: 10.1371/journal.pone.0325981 (PMC12208410; doi:10.1371/journal.pone.0325981)
Supplement: S2 Table — Log10 reduction value was calculated based on the difference in viral titers and viable cell counts following treatment with the control and BAC mixture. (Mean ± SD, n = 3–6). Composition G is a commercially available reagent. “n.t.” indicates not tested, “-“ denotes that no calculable values are available, and “LR” indicates Log Reduction value. (DOCX) [file pone.0325981.s002.docx]

**S2 Table. Virucidal and Bactericidal activity of BAC mixture with alkyl chain lengths of C12, C14 and C16**

|  |  |  | Virucidal Activity | | | | | | | | | | | | | | | | Bactericidal Activity | | | |  |
| --- | --- | --- | --- | --- | --- | --- | --- | --- | --- | --- | --- | --- | --- | --- | --- | --- | --- | --- | --- | --- | --- | --- | --- |
|  |  |  | 10^-4^ M | | | | | | | | | | | | S10^-3^ M | | | | 10^-4^ M | | | |  |
| Sample | C12:C14:C16 composition | CMC (M) | Virus Titer (Log_10_ FFU/mL) | | | | | | | | | LR | | | Virus Titer (Log_10_ FFU/mL) | | | LR | Survivors  (Log_10_ CFU/mL) | | | LR |  |
|  |  |  | 0.5min | | | 5min | | | 10min | | | 0.5min | 5min | 10min | 5min | | | 5min | 5min | | | 5min |  |
| Control |  |  | 6.46 | ± | 0.12 | 6.35 | ± | 0.12 | 6.46 | ± | 0.20 |  |  |  | 6.29 | ± | 0.07 |  | 6.50 | ± | 0.13 |  | |
| A | 1:1:0 | 9.09E-04 | 6.24 | ± | 0.13 | 6.23 | ± | 0.05 | 6.07 | ± | 0.03 | 0.22 | 0.11 | 0.39 | 2.52 | ± | 0.33 | 3.77 | 2.66 | ± | 0.94 | 3.84 | |
| B | 1:1:1 | 3.44E-04 | 6.18 | ± | 0.19 | 6.02 | ± | 0.01 | 5.67 | ± | 0.18 | 0.28 | 0.32 | 0.79 | 1.47 | ± | 0.62 | 4.82 | 1.60 | ± | 0.00 | 4.90 | |
| C | 1:1:3 | 1.85E-04 | 6.04 | ± | 0.26 | 5.56 | ± | 0.17 | 4.76 | ± | 0.16 | 0.42 | 0.79 | 1.70 | 1.28 | ± | 0.32 | 5.01 | 2.24 | ± | 1.04 | 4.26 | |
| D | 1:1:8 | 8.31E-05 | 5.63 | ± | 0.38 | 4.29 | ± | 0.18 | 3.30 | ± | 0.06 | 0.83 | 2.06 | 3.16 | 0.78 | ± | 0.59 | 5.50 | 1.62 | ± | 0.03 | 4.88 | |
| E | 1:1:18 | 7.95E-05 | 5.47 | ± | 0.38 | 4.12 | ± | 0.13 | 3.15 | ± | 0.19 | 0.99 | 2.23 | 3.31 | 0.97 | ± | 0.65 | 5.32 | 1.60 | ± | 0.00 | 4.90 | |
| F | 0:0:1 | 7.43E-05 | 5.37 | ± | 0.27 | 3.68 | ± | 0.14 | 2.51 | ± | 0.26 | 1.09 | 2.66 | 3.95 | 1.01 | ± | 0.38 | 5.28 | 1.60 | ± | 0.00 | 4.90 | |
| G | 61:32:7* | 7.01E-04 | 6.14 | ± | 0.03 | 6.04 | ± | 0.02 | 6.38 | ± | 0.02 | 0.32 | 0.31 | 0.09 | 1.87 | ± | 0.2 | 4.42 | 2.31 | ± | 1.23 | 4.19 | |
| H | 1:0:4 | 6.84E-05 | n.t. | | | 3.77 | ± | 0.47 | n.t. | | | - | 2.58 | - | n.t. | | | - |  | n.t. |  | - | |
| I | 1:2:7 | 7.46E-05 | n.t. | | | 4.39 | ± | 0.42 | n.t. | | | - | 1.96 | - | n.t. | | | - |  | n.t. |  | - | |
| J | 0:2:3 | 7.87E-05 | n.t. | | | 4.47 | ± | 0.43 | n.t. | | | - | 1.87 | - | n.t. | | | - | 1.87 | ± | 0.42 | 4.63 | |
| K | 0:1:4 | 7.88E-05 | n.t. | | | 4.16 | ± | 0.29 | n.t. | | | - | 2.19 | - | n.t. | | | - |  | n.t. |  | - | |
| L | 0:3:2 | 8.66E-05 | n.t. | | | 5.06 | ± | 0.25 | n.t. | | | - | 1.29 | - | n.t. | | | - | 1.45 | ± | 0.00 | 5.05 | |
| M | 2:1:7 | 8.80E-05 | n.t. | | | 4.51 | ± | 0.30 | n.t. | | | - | 1.83 | - | n.t. | | | - |  | n.t. |  | - | |
| N | 2:0:3 | 1.18E-04 | n.t. | | | 4.96 | ± | 0.24 | n.t. | | | - | 1.38 | - | n.t. | | | - | 1.45 | ± | 0.00 | 5.05 | |
| O | 1:2:2 | 1.40E-04 | n.t. | | | 5.52 | ± | 0.11 | n.t. | | | - | 0.82 | - | n.t. | | | - |  | n.t. |  | - | |
| P | 3:0:2 | 1.47E-04 | n.t. | | | 5.60 | ± | 0.27 | n.t. | | | - | 0.75 | - | n.t. | | | - | 2.76 | ± | 0.93 | 3.74 | |
| Q | 2:1:2 | 1.71E-04 | n.t. | | | 5.64 | ± | 0.11 | n.t. | | | - | 0.70 | - | n.t. | | | - | n.t. | | | - | |
| R | 1:3:1 | 2.05E-04 | n.t. | | | 5.74 | ± | 0.03 | n.t. | | | - | 0.61 | - | n.t. | | | - | 1.45 | ± | 0.00 | 5.05 | |
| S | 3:1:1 | 3.97E-04 | n.t. | | | 6.00 | ± | 0.12 | n.t. | | | - | 0.34 | - | n.t. | | | - | 1.84 | ± | 0.04 | 4.66 | |
| T | 2:3:0 | 6.24E-04 | n.t. | | | 6.11 | ± | 0.11 | n.t. | | | - | 0.23 | - | n.t. | | | - | 1.96 | ± | 0.24 | 4.54 | |
| U | 0:1:0 | 7.27E-04 | n.t. | | | 5.87 | ± | 0.17 | n.t. | | | - | 0.47 | - | n.t. | | | - | 1.48 | ± | 0.00 | 5.02 | |
| V | 3:2:0 | 8.11E-04 | n.t. | | | 6.00 | ± | 0.11 | n.t. | | | - | 0.35 | - | n.t. | | | - | 1.89 | ± | 0.31 | 4.61 | |
| W | 1:0:0 | 4.54E-03 | n.t. | | | 6.17 | ± | 0.15 | n.t. | | | - | 0.18 | - | n.t. | | | - | 3.31 | ± | 1.06 | 3.19 | |

Log_10_ reduction value was calculated based on the difference in viral titers and viable cell counts following treatment with the control and BAC mixture. （Mean ± SD, n = 3-6). Composition G is a commercially available reagent. “n.t.” indicates not tested, “-“ denotes that no calculable values are available, and “LR” indicates Log Reduction value.
