# Supplementary material for: Study on the relationship between viral inactivation and alkyl chain length of benzalkonium chloride
Source: PLoS One. 2025 Jun 30;20(6):e0325981. doi: 10.1371/journal.pone.0325981 (PMC12208410; doi:10.1371/journal.pone.0325981)
Supplement: S3 Table — Log10 Reduction is the difference between virus titer of each BAC and the control (Mean, n = 2). (DOCX) [file pone.0325981.s003.docx]

**S3 Table. Virus titer and Log_10_ Reduction of C10 BAC**

| **Concentration (M)** | **Virus Titer [FFU/mL, Log]** | **Log Reduction** |
| --- | --- | --- |
| Control | 6.61 |  |
| 1.60×10^-2^ | 6.47 | 0.14 |
| 3.21×10^-3^ | 6.64 | -0.03 |
| 3.21×10^-4^ | 6.55 | 0.06 |
| 3.21×10^-5^ | 6.70 | -0.09 |
| 3.21×10^-6^ | 6.65 | -0.04 |

Log_10_ Reduction is the difference between virus titer of each BAC and the control (Mean, n=2)
